# Long-term oncologic outcomes of patients with grade group 1 prostate cancer and pT3a disease on final pathology

**DOI:** 10.1038/s41391-026-01138-z

**Published:** 2026-07-17

**Authors:** Mohamad Watfa, Kristina Dortche, Gagan Fervaha, Salim K. Younis, Abdulrahman Al-Bayati, Betty Wang, Rui M. Bernardino, Jesse K. McKenney, Samuel Haywood, Ruben Olivares, Riccardo Autorino, Jihad Kaouk, Christopher J. Weight, Zeyad R. Schwen, Jane K. Nguyen

**Affiliations:** 1 Glickman Urological Institute, Cleveland Clinic, Cleveland, OH USA; 2 Diagnostics Institute, Anatomic Pathology, Cleveland Clinic, Cleveland, OH USA

**Keywords:** Prostate cancer, Prostate cancer

## Abstract

**Background:**

Grade Group 1 (GG1) prostate cancer is biologically indolent and typically managed with active surveillance (AS), yet pT3a pathology is occasionally identified at radical prostatectomy (RP). Its prognostic significance in GG1 disease remains incompletely defined.

**Methods:**

Retrospective cohort of 40 patients with GG1 and pT3a disease on RP (2000–2022), confirmed on central pathologic re-review.

**Results:**

Four patients developed biochemical recurrence (BCR); the cumulative incidence of BCR was 3.2% at 5 years and 10.3% at 15 years. No patient developed metastatic disease or prostate cancer–specific mortality.

**Conclusions:**

GG1 pT3a prostate cancer demonstrates excellent long-term outcomes. Adverse histologic features, not pathological stage alone, drive clinically meaningful risk. Late, stable PSA detectability alone does not signify meaningful recurrence.

## Introduction

Grade Group 1 (GG1) prostate cancer represents the lowest risk tier in the contemporary grading system [1]. It is widely considered an indolent disease, with active surveillance (AS) recommended as the preferred management option by contemporary guidelines [2, 3]. The favorable natural history of GG1 disease has even prompted debate about whether it warrants the cancer designation at all [4].

Despite the overall indolence of GG1 disease, non-organ-confined pathology at radical prostatectomy (RP) is occasionally identified [5]. However, long-term data on patients with GG1 and pT3a disease are limited, and clear guidance for postoperative counseling remains lacking.

Recent evidence has also emphasized that adverse histologic features, such as unfavorable histology, rather than pathological stage alone, drive adverse oncologic outcomes [6], highlighting the need to clarify the prognostic relevance of non-organ-confined disease in otherwise low-grade prostate cancer.

We report the long-term oncologic outcomes of patients with GG1 prostate cancer and pT3a disease (extraprostatic extension (EPE), bladder neck invasion (BNI), or both) confirmed on RP pathology.

## Methods

We retrospectively reviewed institutional pathology archives for RP specimens (2000–2022). Among 12,748 RPs, 799 were pT2 GG1, 56 were recorded as pT3a GG1 (EPE and/or BNI), and no pT3b GG1 cases were identified. The 56 pT3a cases underwent central re-review by a genitourinary pathologist (J.K.N.); 6 were upgraded to GG2, and 10 lacked follow-up, yielding a final cohort of 40 confirmed pT3a GG1 cases. A representative histologic example is shown in Supplementary Fig. S1.

Outcomes were biochemical recurrence (BCR; post-operative PSA ≥ 0.2 ng/mL, confirmed on repeat testing) [7], sub-BCR PSA detectability, metastasis, prostate cancer–specific mortality, and overall survival. Follow-up was measured from RP to the last PSA or death. Cumulative BCR incidence was estimated by Kaplan–Meier methods.

## Results

Baseline characteristics and oncologic outcomes are summarized in Table 1. Of 40 patients, 36 had GG1 on biopsy, and 4 had GG2 downgraded to GG1 on final pathology. pT3a features included EPE alone in 33 patients (83%), BNI alone in 6 (15%), and concurrent EPE/BNI in 1 (2%); pT3a extent was focal in 23 (58%) and non-focal in 17 (42%). Surgical margins were positive in 21 (53%). Only one patient (2022) underwent pre-operative prostate magnetic resonance imaging (MRI); no extracapsular extension (ECE) was identified. No patient received adjuvant radiation.

**Table 1:** Baseline characteristics and long-term oncologic outcomes of patients with Grade Group 1 prostate cancer and pT3a disease on final radical prostatectomy pathology.

Variable	Value (*N* = 40)
Baseline characteristics	
Age at RP, years, median (IQR)	58 (52–64)
Preoperative PSA, ng/mL, median (IQR)	4.8 (3.4–6.0)
Biopsy Grade Group, *n* (%)	
Grade Group 1	36 (90%)
Grade Group 2 (downgraded to GG1 at RP)	4 (10%)
Pre-operative prostate MRI, *n* (%)	
Performed	1 (3%)
Not performed (pre-MRI era)	39 (97%)
Pathologic findings	
pT3a feature, *n* (%)	
Extraprostatic extension only	33 (83%)
Bladder neck invasion only	6 (15%)
Extraprostatic extension and bladder neck invasion	1 (2%)
pT3a extent, *n* (%)	
Focal	23 (58%)
Non-focal	17 (42%)
Nodal pathology, *n* (%)	
pNX	31 (78%)
pN0	9 (22%)
Surgical margin status, *n* (%)	
Negative	19 (47%)
Positive	21 (53%)
Surgical approach, *n* (%)	
Open	20 (50%)
Laparoscopic	5 (13%)
Robot-assisted	15 (37%)
Long-term oncologic outcomes	
Follow-up, years, median (IQR)	15 (5–18)
Biochemical recurrence (BCR), total events	4
Cumulative incidence of BCR at 5 years, % (95% CI)	3.2 (0.5–20.8)
Cumulative incidence of BCR at 10 years, % (95% CI)	10.3 (3.4–28.6)
Cumulative incidence of BCR at 15 years, % (95% CI)	10.3 (3.4–28.6)
Sub-BCR detectable PSA, total events	6
Cumulative incidence of sub-BCR detectable PSA at 10 years, % (95% CI)	6.7 (1.7–24.1)
Cumulative incidence of sub-BCR detectable PSA at 15 years, % (95% CI)	18.7 (8.2–39.5)
Metastatic disease, total events	0
Prostate cancer–specific mortality, total events	0
Other-cause mortality, total events	8

*BCR* biochemical recurrence, *CI* confidence interval, *IQR* interquartile range, *MRI* magnetic resonance imaging, *PSA* prostate-specific antigen, *pN0* no regional lymph node metastasis, *pNX* regional lymph nodes not assessed, *RP* radical prostatectomy.

Data are presented as median (IQR) or *n* (%) unless otherwise indicated. Time-to-event outcomes are reported as total events and Kaplan-Meier cumulative incidence estimates with log-log 95% confidence intervals.

**Fig. 1:**
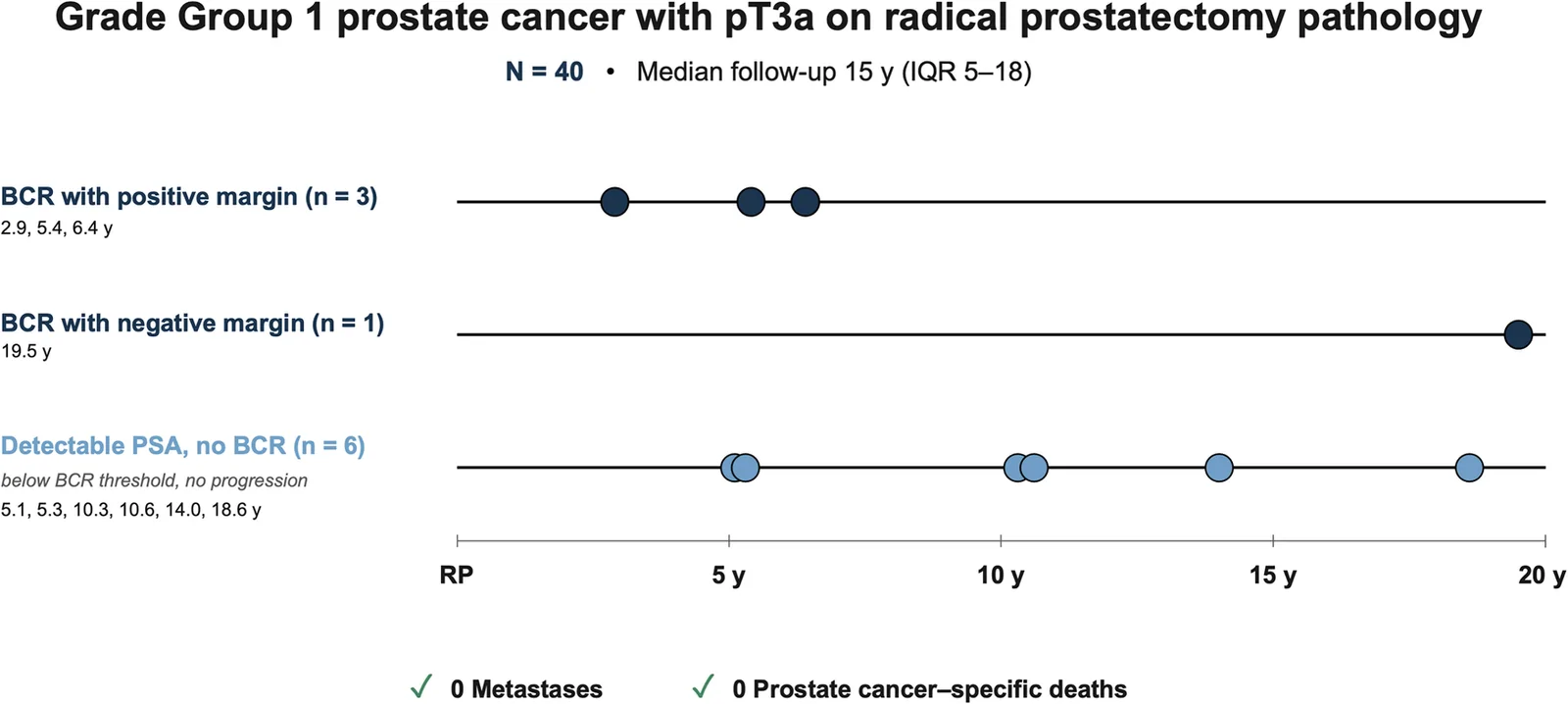
Oncologic outcomes timeline for 40 patients with Grade Group 1 prostate cancer and pT3a disease on final radical prostatectomy pathology. Median follow-up was 15 years (IQR 5–18). BCR occurred in 4 patients: 3 with positive surgical margins (at 2.9, 5.4, and 6.4 years post-RP) and 1 with a negative margin (at 19.5 years). Six patients developed detectable PSA below the BCR threshold at 5.1, 5.3, 10.3, 10.6, 14.0, and 18.6 years post-RP, with all values remaining stable and no subsequent progression. No metastatic disease or prostate cancer–specific mortality was observed. BCR biochemical recurrence (PSA ≥ 0.2 ng/mL, confirmed on repeat testing), RP radical prostatectomy, y years.

Median follow-up was 15 years (IQR 5–18). BCR occurred in 4 patients at 2.9, 5.4, 6.4, and 19.5 years post-RP. The cumulative incidence of BCR was 3.2% at 5 years and 10.3% at both 10 and 15 years. Three patients received salvage radiotherapy, and one received androgen deprivation therapy. The three patients with BCR within 10 years all had positive surgical margins, while the single patient with BCR at 19.5 years had negative margins (Fig. 1). The cumulative incidence of detectable PSA below the BCR threshold was 6.7% at 10 years and 18.7% at 15 years (6 events at 5.1, 5.3, 10.3, 10.6, 14.0, and 18.6 years post-RP). PSA values in these patients remained stable below the BCR threshold, with no progression to BCR. No patient developed metastatic disease or prostate cancer-specific mortality. Eight deaths from other causes were observed during follow-up.

## Discussion

In this cohort with a median follow-up of 15 years, GG1 prostate cancer with pT3a pathology demonstrated highly favorable long-term oncologic outcomes. No patient developed metastatic disease or died from prostate cancer, despite positive margins or non-focal EPE in many. Rezaee et al. similarly observed zero metastasis or cancer-specific mortality in GG1 and EPE disease, though over a median follow-up of only 4 years [5]. Our 15-year median follow-up substantially extends this observation, suggesting that the pT3a stage may not confer meaningful oncologic risk in GG1 tumors.

The observed pattern of BCR, three events within the first 7 years, and a single late event at 19.5 years, is consistent with the indolent natural history characteristic of GG1 disease [4, 8] and aligns with prior evidence showing no significant difference in BCR-free survival between GG1 patients with and without EPE [5]. Moreover, the clustering of recurrences in margin-positive patients supports margin status as the primary driver of recurrence in this setting [9]. The very late single recurrence also aligns with Loeb et al.‘s observation that BCR beyond 15 years is uncommon and generally associated with favorable outcomes [10].

An important aspect of our findings is the pattern of PSA behavior below the BCR threshold. Detectable PSA accumulated gradually over follow-up, with no events in the first 5 years and no subsequent progression to BCR among affected patients, suggesting that low-level PSA detectability may remain stable rather than evolve into clinically meaningful recurrence. In the absence of metastasis or cancer-specific mortality, this is consistent with biologically indolent PSA production, potentially arising from residual benign tissue or non-progressive microscopic disease rather than clinically significant residual carcinoma [7]. Stable sub-BCR PSA plateaus in patients with final GG1 disease should therefore be interpreted cautiously and may not justify reflexive salvage radiation, given the indolent outcomes observed here and in prior series [5, 8].

Our findings support contemporary guideline recommendations favoring AS for most GG1 patients [3, 7], and align with long-term data, including ProtecT, showing low prostate cancer–specific mortality in appropriately selected patients [4, 8, 11]. Yet, some patients still undergo RP because of young age, preference, suspicious imaging findings, high-volume disease or concern for an undersampled higher-grade tumor. ProtecT also underscores the challenge of patient selection, as substantial upgrading and upstaging were observed among men who underwent prostatectomy [11]. This residual uncertainty helps explain why rare post-RP findings such as pT3a GG1 remain relevant in the AS era and create a counseling dilemma directly addressed by our findings. Patients in this scenario may be reassured that routine treatment escalation based on pT3a status alone is not warranted, consistent with evidence that unfavorable histology is the principal driver of adverse oncologic outcomes [6]. Importantly, these findings should not be generalized to higher‑grade tumors, in which adverse histologic features are most prognostic, and the pathological stage may play a more meaningful role [5, 6, 9].

Limitations of this study include the retrospective single-institution design, small sample size, and absence of a pT2 GG1 comparator. No patient had seminal vesicle invasion, limiting extrapolation to pT3b GG1 disease. Additionally, only one patient underwent pre-operative MRI, with no ECE identified, reflecting the predominance of pre-MRI-era cases in our cohort. Therefore, we could not assess the clinical significance of MRI-suspected non-organ-confined features in otherwise GG1 disease, including its relationship to pT3a pathology or its influence on management decisions such as repeat biopsy or treatment selection.

## Conclusions

GG1 prostate cancer with pT3a pathology demonstrates excellent long-term oncologic outcomes, with no metastatic progression or cancer-specific mortality over extended follow-up. These findings underscore that adverse histologic features, such as unfavorable histology, rather than anatomic extent of disease, are the principal drivers of clinically meaningful risk and that late, stable low-level PSA detectability is unlikely to represent clinically significant recurrence in this population. Conservative postoperative management is appropriate in the absence of adverse histologic features or progressive PSA kinetics.

## Supplementary information

Supplementary Figure S1. Representative low-power histologic image demonstrating extraprostatic extension (EPE) in Grade Group 1 prostate cancer.

## Data Availability

The data associated with this paper are not publicly available but are available from the corresponding author upon reasonable request.
